# Arteriovenous blood metabolomics: An efficient method to determine the key metabolic pathway for milk synthesis in the intra-mammary gland

**DOI:** 10.1038/s41598-018-23953-8

**Published:** 2018-04-04

**Authors:** Bing Wang, Huizeng Sun, Xuehui Wu, Linshu Jiang, Le Luo Guan, Jianxin Liu

**Affiliations:** 1 Institute of Dairy Science, College of Animal Sciences; MoE Key Laboratory of Molecular Animal Nutrition, Zhejiang University, Hangzhou, P.R. China; 2 Feed Research Institute, Chinese Academy of Agricultural Sciences, Beijing, 100081 P.R. China; 3 Beijing Key Laboratory for Dairy Cow Nutrition, College of Animal Science and Technology, Beijing University of Agriculture, Beijing, 102206 P.R. China; 4 Department of Agricultural, Food and Nutritional Science, University of Alberta, Edmonton, AB T6G 2P5 Canada

## Abstract

The present study aimed to identify metabolic signature changes of the arteriovenous metabolome and the new metabolites that involved in mammary biological process during milk synthesis. GC/MS-based metabolomics profiling of arteriovenous plasma from 30 lactating dairy cows fed three diets identified a total of 144 metabolites. Phenylalanine and tyrosine, involved in aminoacyl-tRNA biosynthesis and phenylalanine metabolism, were shown higher expression in the artery than in the vein based on both GC/MS and targeted analysis for cows fed both alfalfa hay diet and rice straw diet. Mammary uptake or clearance of citric acid, stearic acid, oleic acid, fructose, β-mannosylglycerate, 4-hydroxybutyrate, and D-talose were significantly correlated with milk performance or feed intake, indicating that these metabolites might be newly identified precursors or indicators of milk synthesis. This comprehensive assessment of metabolic changes in the arteriovenous metabolome will provide a fundamental understanding of the key metabolites involved in milk synthesis and shows implications of how metabolites from arteriovenous plasma across MG are involved in biological processes or physiological functions for milk synthesis. The newly identified metabolites from the present study provide potential new targeted insights into the study of physiological process for milk synthesis in the MG.

## Introduction

Milk from dairy cows is an important daily food for humans due to its high nutritional value. Milk synthesis, which occurs in mammary epithelial cells^1^, is determined by mammary blood flow^2,3^, solute transport^4,5^, net catabolism of milk components precursors^6^, and physiological and nutritional status through regulation of the milk secretory cell number and activity^7,8^. In past decades, nutritional management has been widely applied to improve milk production and quality. However, the mechanisms underlying the regulation of this complicated biological process remain largely undefined.

The increase of mammary plasma flow can greatly improve the arterial nutrient supply to mammary tissue^9^. The arteriovenous difference (AVD) of metabolites is an essential vector and mediator of associated changes in the metabolic process, and this factor has been applied to study intra-tissue metabolic activity and gain insight into normal organ homeostasis and potentially organ-specific pathology^10,11^. Measuring the AVD, mammary blood flow rate, and milk component yield is important for lactation physiology. Thus, the balance between the uptake of precursors for milk production, such as glucose, amino acids (AA), fatty acids, and β-hydroxybutyrate, and output of fat, protein, and lactose in milk can be calculated to estimate the net catabolism of each precursor and understand metabolic changes in the mammary gland (MG)^4,12,13^. Essential AA, including histidine, lysine, threonine, leucine, isoleucine, and valine, can be catabolized to synthesize non-essential AA that are taken up in cases of output deficity^4,14^. Essential AA that are not catabolized are stoichiometrically transferred into protein, providing an understanding of the metabolic progress of milk protein synthesis from the view of essential AA metabolism^4,14^. However, there may be novel metabolites in arteriovenous plasma that also play important roles in milk synthesis. However, knowledge is limited on the uptake and catabolism of these metabolites.

Mass spectrometry has become an increasing powerful tool to analyze metabolites of samples with significant molecular complexity^15^ through non-targeted metabolomics, which can identify biological markers and metabolic pathways to obtain a better understanding of the internal metabolic physiology. Nutritional metabolomics to profile low-molecular weight metabolites can lead to the identification of specific nutritional biomarkers in complex bio-systems research under different nutritional conditions^16^. Plasma is a common biofluid that is widely used for metabolomics and metabolic phenotyping in clinical diagnostics^17,18^ and has also been successfully applied in studies on dairy cows^19–21^.

We hypothesized that un-determined metabolites and milk component precursors, such as AA, fatty acids, glucose, β-hydroxybutyrate, among others, are involved in milk synthesis based on arteriovenous metabolomics, which can be used as markers for intra-mammary metabolic activity. Therefore, the objectives of the present study were to identify different plasma metabolite and metabolic functions in paired arterial vs. venous blood from a circulatory system of MG during lactation using comprehensive untargeted metabolite profiling and investigate the correlation between mammary uptake and milk performance, as well as the correlation between mammary clearance and milk performance.

## Materials and Methods

### Experimental design and blood sample collection

A total of 30 healthy lactating dairy cows (10 cows per group; milk yield = 30.0 ± 3.53 kg, and days in milk = 160 ± 27.8 d; parity = 3.4 ± 1.57; means ± SD) were selected in the present study. The experimental protocols were performed in accordance with the guidelines and regulations approved by the Animal Care Committee, Zhejiang University (Hangzhou, P. R. China). The experiments were designed as previously reported^5^. Briefly, cows were individually fed 3 different diets with identical levels of concentrate and corn silage, and supplemented with either alfalfa hay (23% of DM) plus Chinese wild ryegrass hay (7%) (AH), corn stover (30%, CS), or rice straw (30%, RS) as different forage sources (Table S1). To maintain similar nitrogen levels among the three diets, 1% urea was supplemented in the CS and RS diets, while 1% beet pulp was supplemented in the AH diet. Cows were fed and milked 3 times daily at 0630, 1400, and 2000 h. Diets were fed as total mixed rations.

After a 13-week feeding period, when cows were completely adapted to the dietary treatments, blood sampling was performed according to the procedure shown in Figure S1. The blood samples were collected from the coccygeal artery (arterial plasma sample, W) and superficial epigastric vein (mammary venous plasma sample, R) of 30 cows at 0600 prior to the morning feeding and milking. The cows were standing for at least 10 min prior to blood sampling. A total of 5 mL of blood was drawn using vacuum tubes (Becton Dickinson, Franklin Lakes, NJ, USA) with lyophilized lithium heparin as an anticoagulant. The samples were immediately centrifuged at 3,000 × g for 15 min at 4 °C. Subsequently, the supernatant (plasma) was aliquoted (0.5 mL) into appropriately labeled cryovial tubes and frozen at −80 °C until further analysis.

### Sample preparation for GC-TOF/MS

After thawing on ice for 1 h, the samples were gently mixed well. A mixed sample containing all 57 analyzed samples (30 venous plasma and 27 arterial plasma because 3 artery samples were damaged by mistake) was prepared by mixing approximately 10 μL of each sample into a new 2 mL GC/MS glass vial and used for quality control. Subsequently, the mixed sample and all individual samples were processed using the following procedure: 100 μL of the plasma sample was mixed with 350 μL of methanol and 30 μL of L-2-Chlorophenylalanine (0.1 mg/mL stock in dH_2_O) as an internal standard. After mixing for 10 s and centrifugation at 12,000 rpm for 15 min at 4 °C, the supernatant was transferred (approximately 0.4 mL) to a new 2 mL GC/MS glass vial. After the extracts were dried using a vacuum concentrator at 30 °C for 1.5 h, 60 μL of methoxymethyl amine salt (dissolved in pyridine, final concentration of 20 mg/mL) was added, and after mixing, the vial was gently sealed and incubated at 37 °C for 2 h. Subsequently, 80 μL of bis-trifluoroacetamide (BSTFA, containing 1% trimethylchlorosilane, vol/vol) was added to each sample, and the vial was sealed again and incubated at 70 °C for 1 h. After cooling the sample to the room temperature, 10 μl of FAMEs (saturated mixture of fatty acid methyl esters dissolved in chloroform) was added. Finally, the sample was subjected to detection by GC-TOF/MS (Figure S1).

### Untargeted GC-TOF/MS analysis

GC-TOF/MS analysis was performed using an Agilent 7890 gas chromatograph system coupled with a Pegasus HT TOFMS (LECO, St. Joseph, MI). The system was equipped with a DB-5MS capillary column coated with 5% diphenyl cross-linked with 95% dimethyl polysiloxane (30 m × 250 μm inner diameter, 0.25 μm film thickness; J&W Scientific, Folsom, CA, USA). A 1-μL aliquot of the analyte was injected in split less mode. Helium was used as the carrier gas, with a front inlet purge flow of 3 mL/min and gas flow rate of 20 mL/min. The initial temperature of the column was maintained at 50 °C for 1 min, increased to 330 °C at a rate of 10 °C/min, and subsequently maintained for 5 min at 330 °C. The temperatures of the injection, transfer line, and ion source were 280, 280, and 220 °C, respectively. The energy was −70 eV in electron impact mode. MS data were acquired at a rate of 20 spectra/s after a solvent delay of 366s in full-scan mode with a mass-to-charge ratio (m/z) range of 85–600. An internal standard (L-2-chlorobenzene alanine) was used to ensure consistency among different GC-TOF/MS runs.

### Metabolite profiling

GC-TOF/MS TIC chromatograms of arterial and venous plasma and a detailed data analysis of the metabolites identified from GC-TOF/MS detection is shown in Figure S1. Chroma TOF 4.3X software (LECO Corporation) and the LECO-Fiehn Rtx5 database were used for raw peak extraction, data baseline filtering and calibration of the baseline, peak alignment, deconvolution analysis, peak identification and integration of the peak area according to Kind *et al*.^22^. The RI (retention time index) method was used for peak identification, with the RI tolerance set at 5000. For plasma samples, a numerical simulation method that filled half of the minimum value was used to simulate the missing value of the original data^19^. Subsequently, an interquartile range was used for noise removal and data filtration, and the data were standardized using internal standard normalization methods.

### Metabolite identification

The LECO/Fiehn Metabolomics Library, which provides a similarity value for the accuracy of an identified compound, was used to identify the compounds. If the similarity was greater than 700, then the metabolite identification was considered reliable. If the similarity was less than 200, then the “analyte” was used for metabolite identification. If the similarity was between 200 and 700, then the compound was considered to be a putative annotation. For the GC-Quad Fiehn Lib, we identified derivatives by increasing numbers according to the retention index, e.g., beta-alanine 1 and beta-alanine 2 (for the derivatives with no one or two TMS-groups derivatizing the primary amino group), according to Kind *et al*.^22^.

### Differential metabolites identification

Differential metabolites (DMs) were identified based on pair-wise comparisons between the artery and vein (W vs. R) under each diet. The characteristics of the metabolites were analyzed using multivariate statistics in the SIMCA-P^+^ 13.0 software package (Umetrics, Umea, Sweden). Principal component analysis (PCA), partial least-square discriminant analysis (PLS-DA) and orthogonal PLS-DA (OPLS-DA) were used to identify the effects of the blood vessel and diet on the metabolites. PCA enabled visualization of the data to identify the inherent grouping of samples as a result of similarities and difference of the metabolites. Moreover, a supervised PLS-DA model was applied and validated using 10-fold permutation tests to obtain a higher level of group separation and better understanding of the variables responsible for classification. Further, OPLS-DA was used to obtain maximal covariance between the measured data and response variable and identify the significantly different metabolites between the two sample groups. To refine this analysis by variable selection, the first principal component of OPLS-DA, variable importance projection (VIP), was used to evaluate differences. Variables with VIP > 1.0 were defined as significantly changed metabolites. These metabolites were subsequently assessed using Student’s T test (T-test). The fold-change (FC) value of each metabolite was calculated by comparing the mean value of the peak area obtained from any comparison, and the log_2_FC value was used to indicate the specific variable quantity in the comparison. If the p-value < 0.05, with |log_2_FC| > 0.2 and q-value < 0.05 as cut-offs, then the variables were considered different between any two comparison groups. The Q-value was used to adjust the false discovery rate in the comparison between artery and vein^23^. The DMs were identified and validated using various databases, including the Kyoto Encyclopedia of Genes and Genomes (KEGG), Human Metabolome Database (HMDB), Bovine Metabolome Database (BMDB), PubChem Compound, Chemical Entities of Biological Interest (ChEBI), Japan Chemical Substance Dictionary Web (NIKKAJI), and Chemical Abstracts Service (CAS).

### Quantification analysis of the glucose and amino acid concentrations

The plasma AA concentration was analyzed with norleucine as an internal standard in an Automatic AA Analyzer (Hitachi High-technologies Corporation, Tokyo, Japan). To measure the plasma glucose levels, plasma was pretreated with ice-cold sulfosalicylic acid (50 g/L) at a ratio of 1:4 (v/v) to precipitate protein^24^, followed by centrifugation at 8,320 × g for 30 min at 4 °C. The supernatant was filtered through 0.45-μm and 0.22-μm nylon syringe filter units (Fisher Scientific, Pittsburgh, PA) and placed in microcentrifuge tubes (catalog no. 05-664-34, Fisher Scientific). The plasma glucose concentrations were subsequently measured using an Auto Analyzer 7020 instrument (Hitachi High-technologies) and commercial colorimetric kit (DiaSys Diagnostics Systems GmbH, Frankfurt, Germany)^25^.

### Functional analysis of differential metabolites

To explore potential metabolic pathways across MG during milk synthesis, the identified DMs or metabolites whose mammary uptake or clearance were significantly correlated with milk performance were imported into the online analysis platform Metaboanalyst (http://www.metaboanalyst.ca/) to identify the associated pathways according to Xia *et al*.^26^. The analysis used the *Bos taurus* (cow) pathway library and integrated global test pathway enrichment analysis as well as relative-betweenness centrality pathway topology analysis. All of the matched pathways according to the p-values from pathway enrichment analysis and pathway impact values from pathway topology analysis are shown in the metabolome view.

### Statistical correlation analysis

To obtain a more complete picture of the arterial and venous plasma metabolome and detect undefined or new metabolites associated with the milk synthesis in MG, the correlations between mammary uptake, clearance of metabolites and corresponding milk performance at the 14^th^ week were calculated using Pearson’s Product-Moment correlation analysis. The performance data, including dry matter intake, milk production and components, were obtained from previously published data^2^ as summarized in Table S2. The calculation of mammary plasma flow, mammary uptake, and clearance of metabolites was based on the AVD following the description of Hanigan *et al*.^27^:

$$\begin{array}{rcl}{\rm{AVD}} & = & \mathrm{arterial}-\mathrm{venous}\\ {\rm{Mammary}}\,{\rm{plasma}}\,{\rm{flow}} & = & ({\rm{milk}}\,{\rm{phenylalanine}}+{\rm{tyrosine}})\,({\rm{g}}{\rm{/}}{\rm{d}})\times 0.965/\\  &  & [{\rm{AV}}\,{\rm{difference}}\,{\rm{of}}\,({\rm{phenylalanine}}+{\rm{tyrosine}})\,({\rm{g}}{\rm{/}}{\rm{L}})]\\ {\rm{Mammary}}\,{\rm{uptake}} & = & {\rm{AVD}}\times {\rm{mammary}}\,{\rm{plasma}}\,{\rm{flow}}\\ {\rm{Mammary}}\,{\rm{clearance}} & = & ({\rm{AVD}}\times {\rm{mammary}}\,{\rm{plasma}}\,{\rm{flow}})/{\rm{venous}}\end{array}$$

A correlation coefficient (r) and the significance threshold of |r| ≥ 0.50 and p-value < 0.01 were applied in the correlation between mammary uptake, clearance of metabolites and milk performance. The correlations were confirmed after scatter plotting each pair of correlated integrals with R-3.2.2 statistical software^28^.

Quantitative analysis of the data was performed using a pair-wise comparison between the artery and vein using T-test under each diet. The feed intake and milk performance were analyzed as a completely randomized design using PROC MIXED of SAS. A completely randomized block design was used, with diet as the fixed effect and cows within each diet as the random effect for the feed intake and milk performance. The results are reported as the least squares means. The statistical significance of the main effects was declared at P < 0.05.

## Results

### Identification of metabolites and validation of selected metabolites

In total, 444 peaks were detected from all plasma samples. Based on the LECO/Fiehn Metabolomics Library, the majority of the peaks were endogenous metabolites. From 444 detected peaks, only 144 defined metabolites with peak similarities >400 were subjected to downstream analysis (Supplemental Dataset 1).

### Differently abundant metabolites between arterial and venous plasma within three diets

**Figure 1:**
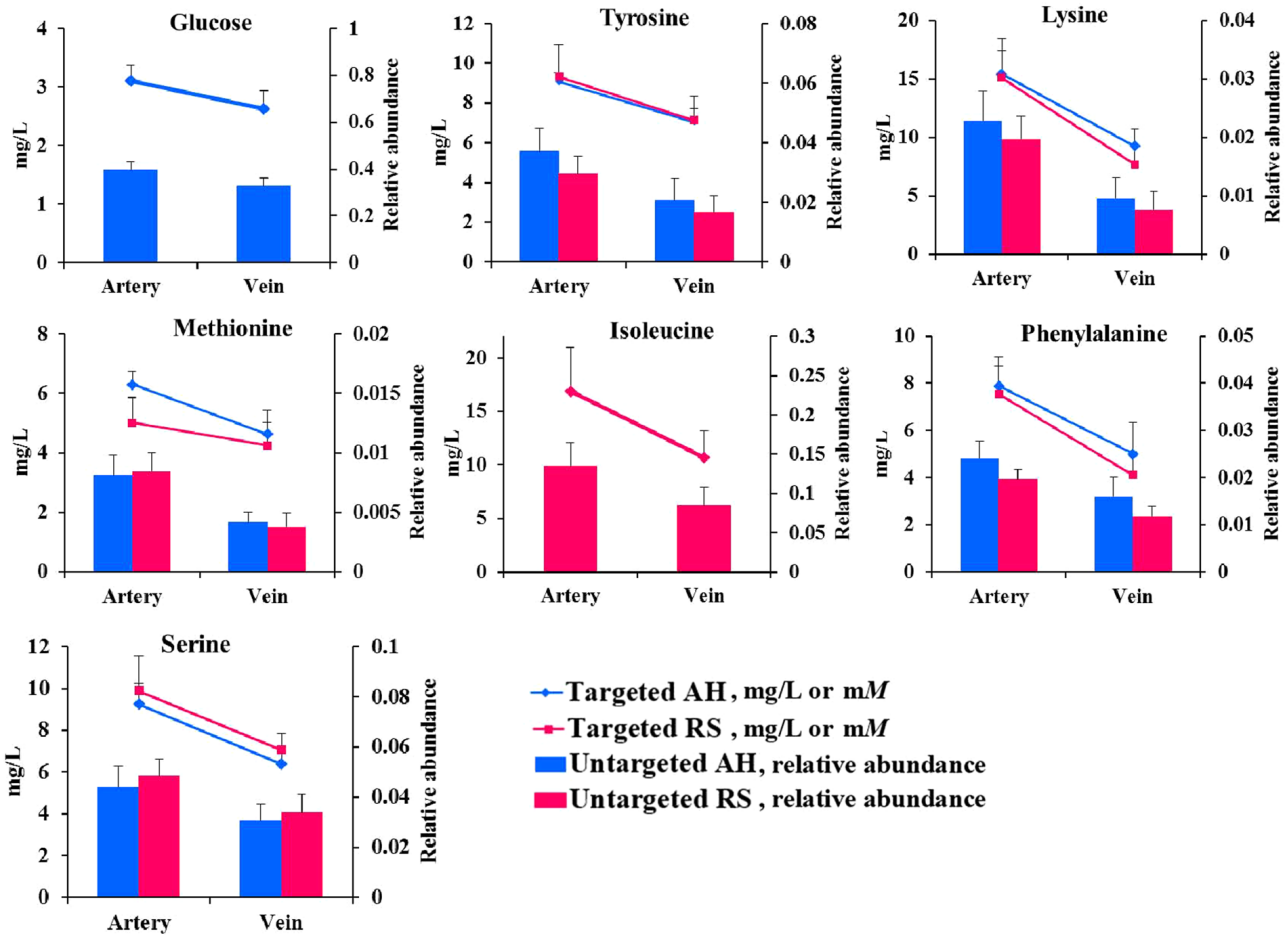
Quantitative concentration (targeted) and relative abundance (untargeted) of glucose, serine, isoleucine, phenylalanine, tyrosine, methionine, and lysine in the artery and vein. Note: all targeted analyses showed significant differences between the artery and vein within the alfalfa hay (AH) and rice straw (RS) diet (P < 0.001). The error bars indicate the standard error.

The PCA, PLS-DA, and OPLS-DA plots of metabolites profiles detected using GC-TOF/MS showed significantly separated clusters between the arterial and venous plasma samples within three dietary conditions (Table S3, Figures S2–S4). Using untargeted GC/MS analysis, 10, 1, and 10 significantly DMs (VIP > 1, P < 0.05, and q < 0.05) were identified between arterial vs. venous plasma when cows fed AH (W-AH vs. R-AH), CS (W-CS vs. R-CS), and RS (W-RS vs. R-RS), respectively, in which all the DMs had higher relative abundance in artery than in vein (Table 1). Out of the 10 significantly DMs detected in AH-fed cows (W-AH vs. R-AH), 8 metabolites belong to AA and amino acid conjugates including ornithine (log_2_FC = 0.82), tyrosine (log_2_FC = 0.83), lysine (log_2_FC = 1.25), methionine (log_2_FC = 0.96), allothreonine (log_2_FC = 0.53), 5-oxoproline (log_2_FC = 0.76), phenylalanine (log_2_FC = 0.59), and serine (log_2_FC = 0.53), and 2 metabolite belongs to carbohydrates and carbohydrate conjugates including glucose (log_2_FC = 0.28) and 3-hydroxybutyric acid (log_2_FC = 0.77). Under CS diet, the relative abundances of 3-hydroxybutyric acid (log_2_FC = 0.72) was higher in artery than in vein. Similarly, out of the 10 DMs from cows fed with RS, ornithine (log_2_FC = 0.91), tyrosine (log_2_FC = 0.84), lysine (log_2_FC = 1.38), isoleucine (log_2_FC = 0.66), methionine (log_2_FC = 1.15), allothreonine (log_2_FC = 0.55), 5-oxoproline (log_2_FC = 0.76), phenylalanine (log_2_FC = 0.74), and serine (log_2_FC = 0.53) were involved in the AA and amino acid conjugates, 3-hydroxybutyric acid (log_2_FC = 0.83) were involved in carbohydrates and carbohydrate conjugates. Quantitative analysis for glucose and AA using standard substance based on specific equipment validated a higher artery concentration of 7 metabolites, including glucose (FC = 1.18), serine (FC = 1.45), lysine (FC = 1.67), phenylalanine (FC = 1.58), tyrosine (FC = 1.29), and methionine (FC = 1.36), than in the vein (p < 0.01) under AH diet, which was similar to the FC results using untargeted GC/MS analysis (Fig. 1). Under RS diet, tyrosine (FC = 1.31), lysine (FC = 1.96), isoleucine (FC = 1.58), phenylalanine (FC = 1.84), serine (FC = 1.40), and methionine (FC = 1.18), than in the vein (P < 0.01), which was similar to the FC results using untargeted GC/MS analysis (Fig. 1).

**Table 1:** Identification of significantly different abundance metabolites between arterial (W) and venous (R) plasma when cows were fed alfalfa hay (AH), corn stover (CS), or rice straw (RS) as forage source diets (VIP > 1, p-value < 0.05, and q-value < 0.05).

Metabolites name	Similarity	R.T.	Mass	VIP	p-value	q-value	log_2_FC^1^
*W-AH vs. R-AH*							
Ornithine	957	16.938	142	2.027	<0.001	0.005	0.82
Glucose	936	17.958	319	4.032	<0.001	0.006	0.28
Tyrosine	914	18.185	218	2.040	<0.001	0.005	0.83
3-Hydroxybutyric acid	900	8.748	147	12.156	<0.001	<0.001	0.77
Lysine	878	18.024	174	1.951	<0.001	<0.001	1.25
Methionine	838	13.566	176	1.083	<0.001	<0.001	0.96
Allothreonine	837	11.855	117	1.623	0.002	0.046	0.53
5-Oxoproline	833	13.602	156	3.476	<0.001	0.012	0.76
Phenylalanine	828	14.856	218	1.482	<0.001	0.005	0.59
Serine	727	11.516	204	1.835	0.001	0.024	0.53
*W-CS vs. R-CS*							
3-Hydroxybutyric acid	900	8.748	147	9.980	<0.001	0.013	0.72
*W-RS vs. R-RS*							
Ornithine	957	16.938	142	1.939	<0.001	0.001	0.91
Tyrosine	914	18.185	218	1.695	<0.001	0.002	0.84
3-Hydroxybutyric acid	900	8.748	147	11.403	<0.001	<0.001	0.83
Lysine	878	18.024	174	1.722	<0.001	<0.001	1.38
Isoleucine	872	10.628	158	3.361	<0.001	0.012	0.66
Methionine	838	13.566	176	1.060	<0.001	<0.001	1.15
Allothreonine	837	11.855	117	1.599	0.002	0.031	0.55
5-Oxoproline	833	13.602	156	3.439	<0.001	<0.001	0.76
Phenylalanine	828	14.856	218	1.395	<0.001	<0.001	0.74
Serine	727	11.516	204	1.858	<0.001	0.004	0.53

^1^FC = fold change (W/R).

**Figure 2:**
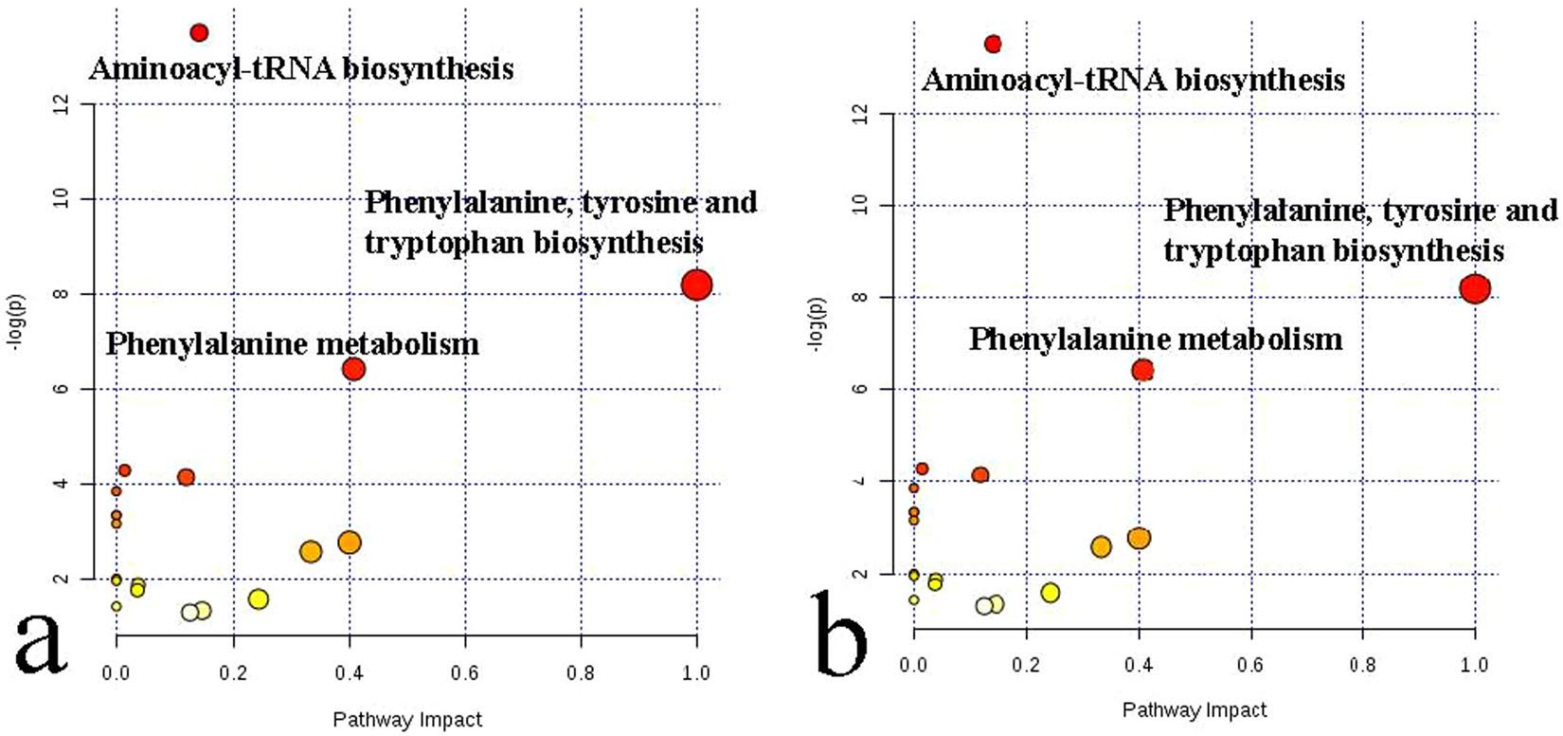
Metabolome view map of the metabolites identified in the comparison between the artery and vein within the alfalfa hay (**a**) and rice straw (**b**) diet. The x-axis represents the pathway impact and y-axis represents pathway enrichment. Larger sizes and darker colors represent higher pathway enrichment and higher pathway impact values, respectively.

The metabolome view map revealed the enriched pathways based on KEGG analysis (p < 0.05) for DMs identified between artery and vein within each diet (Fig. 2). In the light of the fact that the pathways with a high impact value and p-value were regarded as potential pathways. The pathway of phenylalanine, tyrosine and tryptophan biosynthesis, aminoacyl-tRNA biosynthesis, and phenylalanine metabolism were enriched from DMs between artery and vein within AH (Fig. 2a) and RS (Fig. 2b) diets.

### Relationship between feed intake, milk performance and mammary uptake or clearance of metabolites

**Figure 3:**
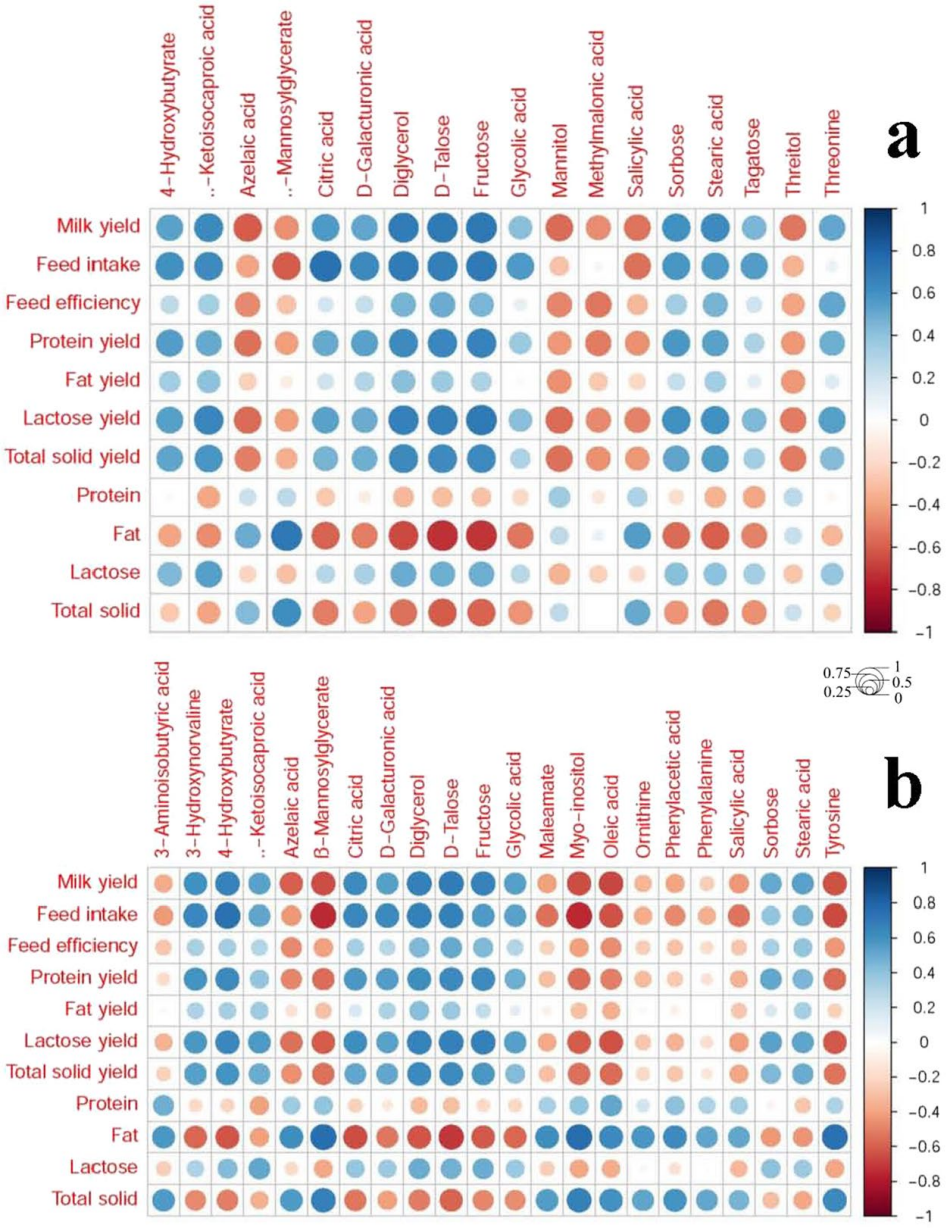
Heatmap of the linear correlation between the characteristics of milk performance and mammary uptake (**a**) or clearance (**b**) of some metabolites. The circle size was correlated with the color. The bigger circle size means the deeper color. The circle size relates with the absolute value of correlation (|R|). The bigger circle size, the higher correlation. Mammary uptake = arteriovenous difference × mammary plasma flow; Mammary clearance = (arteriovenous difference × mammary plasma flow)/venous. The protein, fat and lactose refer to % components in milk. The mammary plasma flow was according to Wang *et al*.^2^

The relationship between the mammary uptake or clearance of metabolites and selected milk performance characteristics were further subjected to a correlation analysis through integration of the linear regression models (Fig. 3, Tables S4, S5). The abundance of metabolites, including diglycerol, stearic acid, citric acid, ß-ketoisocaproic acid, glycolic acid, salicylic acid, fructose, 4-hydroxybutyrate, azelaic acid, and sorbose, was mutually significantly correlated with milk performance between mammary uptake and clearance. Specifically, mammary uptake of stearic acid was positively correlated with feed intake (r = 0.62, P < 0.01), yield of milk (r = 0.57, P < 0.01), protein (r = 0.53, P < 0.01), lactose (r = 0.60, P < 0.01), and total solids (r = 0.55, P < 0.01), but negatively correlated with the milk fat content (r = −0.60, P < 0.01). However, mammary clearance of stearic acid was positively correlated with the yield of milk (r = 0.54, P < 0.01) and lactose (r = 0.53, P < 0.01). Mammary uptake of fructose and citric acid was positively correlated with the milk yield (r = 0.72, P < 0.01) and feed intake (r = 0.75, P < 0.01), respectively. Mammary uptake of azelaic acid and β-mannosylglycerate was negatively correlated with milk yield (r = −0.60, P < 0.01) and feed intake (r = −0.60, P < 0.01), respectively. Mammary uptake of methylmalonic acid was negatively correlated with feed efficiency (r = 0.52, P < 0.01). Mammary clearance of oleic acid and β-mannosylglycerate showed the highest negative correlation with the milk yield (r = −0.67, P < 0.01) and feed intake (r = −0.74, P < 0.01). Mammary clearance of D-talose and 4-hydroxybutyrate showed the highest positive correlation with the milk yield (r = 0.69) and feed intake (r = 0.75, P < 0.01). Mammary uptake and mammary clearance of sorbose were both positively correlated with the milk yield (r = 0.60 and r = 0.51, P < 0.01).

**Figure 4:**
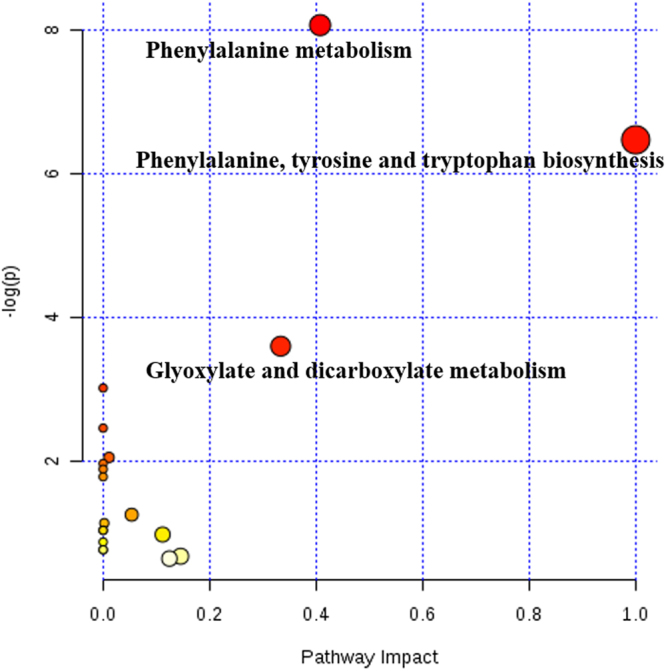
Metabolome view map of the identified significantly correlated metabolites in the comparison between mammary uptake and clearance and milk performance. The x- axis represents the pathway impact and y-axis represents pathway enrichment. Larger sizes and darker colors represent higher pathway enrichment and higher pathway impact values, respectively.

Three key metabolic pathways, including phenylalanine metabolism; phenylalanine, tyrosine and tryptophan biosynthesis; and glyoxylate and dicarboxylate metabolism, were enriched because the uptake and clearance of their metabolites were significantly correlated with milk performance (Fig. 4).

## Discussion

**Figure 5:**
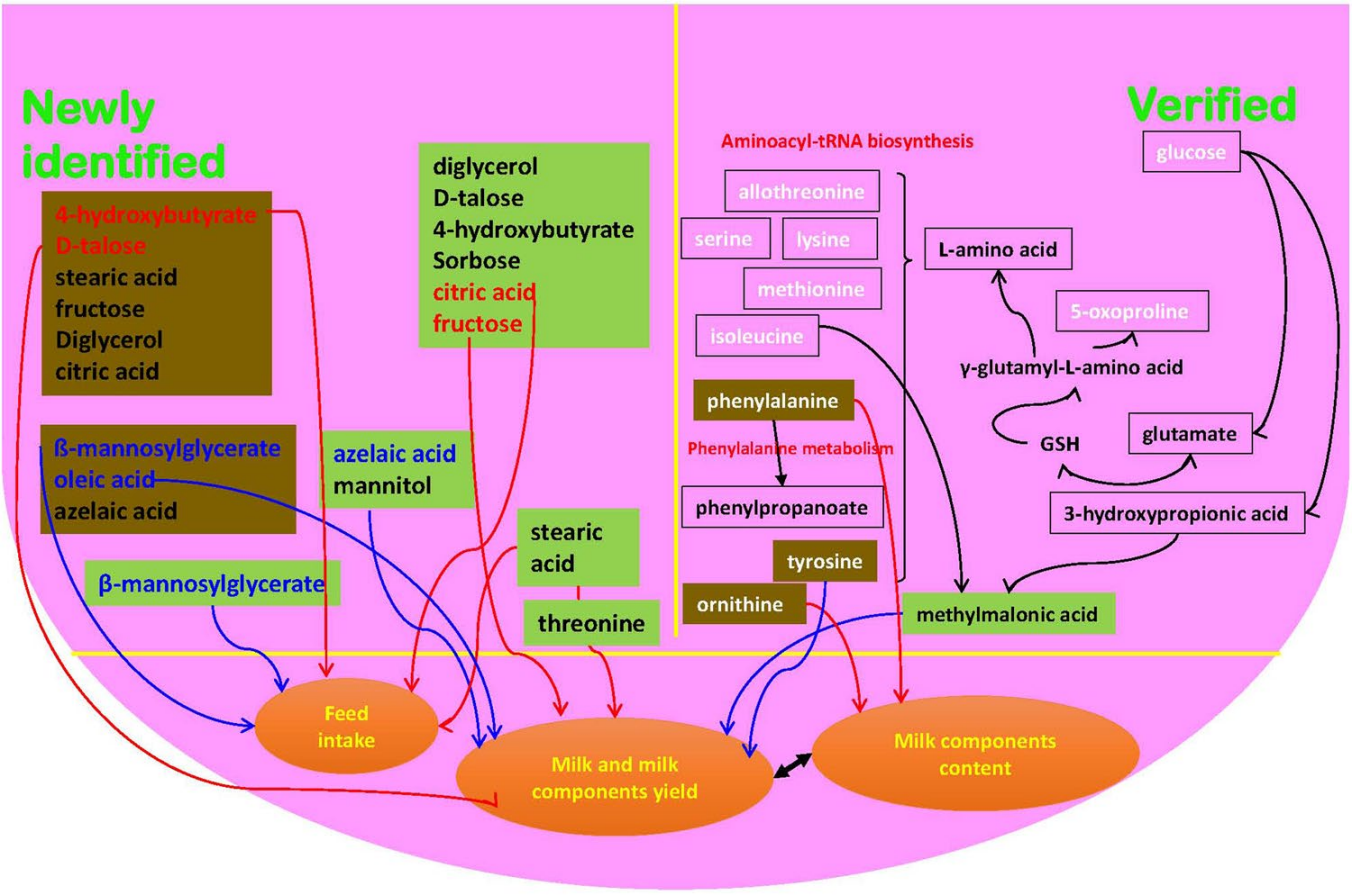
A working hypothesis of the association between critical metabolites across the mammary gland for milk synthesis. Metabolites in the right part in white denote a quantitatively significant difference between the artery and vein within the three diets. Metabolites in the green box and brown box denote a significant correlation between mammary uptake and the clearance of metabolites and milk performance, respectively. The red line denotes the significantly positive correlation between these 2 items, while the blue line denotes the significantly negative correlation between these 2 items. The metabolites in red indicate the highest positive correlation with the milk yield or feed intake; and those in blue indicate the highest negative correlation with the milk yield or feed intake. The black double-sided arrow indicates that the correlation with the metabolites was always the opposite between these 2 items.

Identification of DMs between the artery and vein is a useful approach to explore the internal metabolic process of intra-tissue metabostasis^10^. The enriched pathways based on identified DMs between the artery and vein are primarily related to aminoacyl-tRNA biosynthesis; phenylalanine, tyrosine and tryptophan biosynthesis; and phenylalanine metabolism. Phenylalanine, tyrosine and tryptophan biosynthesis is not found in mammary glands^6^; thus, this pathway was removed from key pathways associated with milk synthesis. However, phenylalanine is a different metabolite between the artery and vein and cannot be synthesized from other metabolites, confirming the importance of essential AA supplied from the artery as milk component precursors for lactation in MG^13^. In addition, higher concentration of essential AA, glucose, and β-hydroxybutyrate acid were detected in the artery compared to the vein within the three diets, which was also confirmed using targeted and un-targeted methods, and the artery provides glucose and AA for milk component synthesis^4^. These findings are consistent with previous studies on AA utilization and metabolism in the MG of dairy cows^4,13^. In addition, the γ-glutamyl cycle involves the transport of AA from the extracellular to the intracellular environment to fulfill the requirements of the AA transport system^29,30^. Allothreonine and serine are involved in the aminoacyl-tRNA biosynthesis pathway and show a direct relationship with glycine and glutamate anabolism and catabolism. These observations were consistent with recent studies reporting that the most essential AA and several non-essential AA are utilized as precursors for milk protein synthesis or the synthesis of other AA or undergo catabolism as a result of transamination or deamination^4,12^. Furthermore, the transamination function was validated by the simultaneously increased AVD and mammary uptake of phenylalanine and tyrosine^31^ and enrichment of the phenylalanine metabolism pathway. The AVD of phenylalanine and tyrosine across the MG was constant and therefore considered to be an internal marker for determining blood flow^14^. Phenylalanine and tyrosine involved in phenylalanine metabolism were significantly different between the artery and vein, and a significant correlation between mammary clearance of phenylalanine and tyrosine with the milk protein content or milk yield was also observed, which might confirm the important role for these AAs in milk synthesis^32^. In addition, essential AA, such as isoleucine, leucine, and threonine, can affect protein synthesis by regulating mTOR signaling^33^. Thus, the findings of the present study suggest that essential AAs can directly or indirectly affect milk protein synthesis. Based on key arteriovenous plasma DMs, pathways, and their correlations with milk parameters, an integrated map and network associated with the milk synthesis process is proposed (Fig. 5). The key metabolites involved in these pathways were lysine, methionine, isoleucine, phenylalanine, and tyrosine, consistent with previous findings on AA metabolism in MG^2,4,12,13^. However, integrated identification of AA and their relationships or interactions with other metabolites are needed to obtain a better understanding of AA utilization in MG for milk synthesis.

Currently, the overall metabolites from both artery and vein across the MG in dairy cows have not been studied, particularly for their AVD, which may help to identify newly un-determined information associated with milk synthesis based on metabolites. The AVD can affect mammary uptake of AA, such as leucine^2^, lysine, methionine, and arginine^34^, and further affect milk component synthesis in MG. However, only focusing on AA may be biased due to the number of metabolites and pathways involved in the milk secretion process^29^. In addition, clearance rates are a function of unidirectional transport rates, explaining the mammary net uptake of EAA in cows with small to moderate perturbations of supply^13^, but this factor does not completely represent the biology of the system and fails when challenged with data from animals in which large perturbations in the system have been imposed^12^. However, the net mammary uptake of EAA reduced prediction errors for the estimation of mammary activity and utilization of AA in the mammary gland^12,35^. Thus, the close correlation between clearance and uptake indicate that the use of both of mammary uptake and clearance together can represent the actual mammary activity. The newly identified metabolites based on mammary uptake and clearance were significantly correlated with the yield and composition of milk and feed intake (Fig. 5), which might be a suggestion for routinely measuring these new metabolites in further studies on mammary physiology. Normal milk contains relatively large amounts of citric acid^36,37^ that is taken up from the arterial supply or catabolized from other substances, such as glucose, pyruvic acid, maltose or glycogen, in the MG tissue. Stearic acid (C18:0) is the most abundant fatty acid from diets available to dairy cows and plays a more prominent role in milk production than C16:0 due to the role of MG in desaturating C18:0 to oleic acid^38^. The importance of exogenous stearic acid and its metabolism in lactating cows may reflect the role of this compound in directly or indirectly regulating triglyceride synthesis^39^. Thus, the positive correlation of the mammary uptake of stearic acid with the milk yield and negative correlation with the milk fat content might confirm the advantageous function of stearic acid in lipid metabolism. Oleic acid is the principal unsaturated acid in bovine milk^40^, and an estimated 30–40% of oleic acid is synthesized in mammary tissue by stearoyl desaturase on absorbed exogenous stearic acid^39^. Oleic acid has been implicated in milk fat synthesis^38^, which was also confirmed by the increased milk content of *trans*-10-18:1 when cows were fed sunflower seeds with high concentration of oleic acid^41^. Thus, the negative correlation of mammary clearance of oleic acid with the milk yield and feed intake but positive correlation with the milk fat content might indicate that the oleic acid plays an important role in regulating milk fat synthesis.

Unlike glucose, fructose cannot be converted to lactose in MG tissue, but is oxidized in the udder to metabolic intermediates that lead to the synthesis of milk protein, fat, citrate and CO_2_^42^. Thus, fructose uptake in MG may contribute to the energy supply and generation of milk composition precursors through its catabolism. Increments in the blood levels of glucose and insulin were significantly suppressed after administration of sorbose to rats compared with the non-addition of sorbose^43^, indicating its role as an energy carrier analogous with glucose. Thus, the positive correlation between the mammary uptake of sorbose and milk yield may reflect the role of sorbose in energy supply. Azelaic acid has the oxyradical-scavenging activity in biological systems^44^; thus, its increased uptake in MG indicates the abnormal status of mammary tissue resulting in lower milk synthesis. Frobish and Davis^45^ proposed that an accumulation of methylmalonic acid in the blood of cows could increase the amount of propionate presented to the liver for metabolism, which might subsequently increase gluconeogenesis. In the present study, however, less methylmalonic acid was detected in the artery compared to the vein, and a significant negative correlation between AVD or mammary uptake of methylmalonic acid and milk or milk protein yield was observed. It was reported that the low-fat milk syndrome is not caused by accumulation of methylmalonic acid^46^, thus the methylmalonic acid is not the precursor for milk fat synthesis. Moreover, a highly negative correlation between mammary plasma flow and mammary clearance of methylmalonic acid with a strong slope was observed, which might indicate that methylmalonic acid is a waste product of milk synthesis. The roles of the other metabolites listed in Fig. 5, such as 4-hydroxybutyrate, D-talose, β-mannosylglycerate, diglycerol, and mannitol, have not been reported in association with milk performance or the direct/indirect regulation of animal metabolic physiology. Thus, additional studies are needed to confirm the biological functions of these metabolites in MG. On the other hand, a future direction for the study of the comparisons between different diets on the metabolome in artery, vein, or AVD should be included to real the dietary effects on the biological changes in MG.

## Conclusion

Arteriovenous metabolomics is an efficient method to identify the undetermined key metabolites and metabolic pathways of milk synthesis in intra-MG. DMs, such as phenylalanine and tyrosine in the pathways of phenylalanine metabolism, glucose, tyrosine, lysine, methionine, and serine between the artery and vein were identified using both untargeted and targeted analyses. These DMs can serve as potential primary biomarkers for metabolic progress in MG. The mammary uptake or clearance of citric acid, stearic acid, oleic acid, fructose, β-mannosylglycerate, 4-hydroxybutyrate, D-talose, sorbose, among others, was significantly correlated with milk performance or feed intake, and these factors might represent newly identified precursors, regulatory factors, or indicators for milk synthesis or the feed intake of dairy cows. Overall, the present study provides a fundamental understanding of the arteriovenous metabolome across MG and shows implications of how metabolites from arteriovenous plasma across MG are involved in biological processes or physiological functions for milk synthesis. The results from the present study provide useful insights into the physiological process for milk synthesis in the MG.

## Electronic supplementary material

Supplemental Tables and Figures

Supplemental Dataset 1

Supplemental Dataset 2
