# Supplementary material for: Arteriovenous blood metabolomics: An efficient method to determine the key metabolic pathway for milk synthesis in the intra-mammary gland
Source: Sci Rep. 2018 Apr 4;8:5598. doi: 10.1038/s41598-018-23953-8 (PMC5884783; doi:10.1038/s41598-018-23953-8)
Supplement: Supplementary file 1 — Supplemental Tables and Figures [file 41598_2018_23953_MOESM1_ESM.doc]

**Arteriovenous blood metabolomics: An efficient method to determine the key metabolic pathway for milk synthesis in the intra-mammary gland**

Bing Wang^1,2^, Huizeng Sun^1^, Xuehui Wu^1^, Linshu Jiang^3^, Le Luo Guan^4,*^, Jianxin Liu^1,*^

^1^Institute of Dairy Science, College of Animal Sciences; MoE Key Laboratory of Molecular Animal Nutrition, Zhejiang University, Hangzhou;

^2^Feed Research Institute, Chinese Academy of Agricultural Sciences, Beijing 100081, P.R. China;

^3^Beijing Key Laboratory for Dairy Cow Nutrition, College of Animal Science and Technology, Beijing University of Agriculture, Beijing 102206, P.R. China;

^4^Department of Agricultural, Food and Nutritional Science, University of Alberta, Edmonton, AB, Canada, T6G 2P5.

*Corresponding authors:

Dr. Leluo Guan: Tel: 1-780-4922480; Fax: 1-780-4924265; E-mail: [lguan@ualberta.ca](mailto:lguan@ualberta.ca)

Dr. Jianxin Liu: Tel: 86-571-88982097; Fax: +86-571- 88982930; E-mail: [liujx@zju.edu.cn](mailto:liujx@zju.edu.cn)

**Table S1. Ingredients and nutrient composition of the total mixed ration containing alfalfa hay (AH), corn stover (CS), or rice straw (RS) as the main forage.**

|  | Treatment | | |
| --- | --- | --- | --- |
| Items | AH | CS | RS |
| Dietary ingredient, % of DM | |  |  |
| Ground corn grain | 27.0 | 27.0 | 27.0 |
| Wheat bran | 5.10 | 5.10 | 5.10 |
| Soybean meal | 12.7 | 12.7 | 12.7 |
| Cottonseed meal | 4.30 | 4.30 | 4.30 |
| Beet pulp | 1.00 | 0.00 | 0.00 |
| Corn silage | 15.0 | 15.0 | 15.0 |
| Alfalfa hay | 23.0 | 0.00 | 0.00 |
| Chinese wild grass hay | 7.00 | 0.00 | 0.00 |
| Corn stover | 0.00 | 30.0 | 0.00 |
| Rice straw | 0.00 | 0.00 | 30.0 |
| Urea | 0.00 | 1.00 | 1.00 |
| Premix^1^ | 4.90 | 4.90 | 4.90 |
| Nutrient composition, % of DM | |  |  |
| Organic matter | 92.3 | 92.3 | 90.1 |
| Crude protein | 16.8 | 16.3 | 16.2 |
| Neutral detergent fiber | 31.8 | 36.7 | 36.5 |
| Acid detergent fiber | 17.5 | 20.9 | 20.2 |
| Ca | 0.81 | 0.62 | 0.56 |
| P | 0.46 | 0.47 | 0.39 |
| NE_L_, MJ/kg | 6.57 | 6.07 | 5.98 |

^1^Formulated to provide (per kilogram of DM): 174 g of zeolite powder, 1.25 g of yeast, 25 g of mold adsorbent (Solis Mos, Novus International Inc., St. Charles, MO), 21.44 g of KCl, 41.25 g of MgO, 150 g of Salt, 187.5 g of NaHCO_3_, 84 g of Ca, 15 g of P, 125,000 IU of vitamin A, 750,000 IU of vitamin D3, 937.5 IU of vitamin E, 1750 mg of Zn, 17.5 mg of Se, 28.75 mg of I, 375 mg of Fe, 15 mg of Co, 556.5 mg of Mn and 343.75 mg of Cu.

^2^NE_L_ = Net energy for lactating, calculated based on the Ministry of Agriculture of P. R. China recommendations (MoA, 2004).

**Table S2**. Lactation performance in dairy cows fed the total mixed ration containing alfalfa hay (AH), corn stover (CS), or rice straw (RS) as the main forage.

|  | Treatment | | |  |  |
| --- | --- | --- | --- | --- | --- |
| Items | AH | CS | RS | SEM | *P*-value |
| Dry matter intake, kg/d | 17.6 | 16.9 | 17.0 | 0.24 | 0.15 |
| Dry matter digestibility, % | 57.9^a^ | 53.0^b^ | 49.0^c^ | 0.95 | <0.01 |
| Milk yield, kg/d | 24.1^a^ | 18.4^b^ | 19.8^b^ | 0.72 | <0.01 |
| Milk composition, % |  |  |  |  |  |
| Milk fat, % | 3.79 | 4.24 | 4.28 | 0.208 | 0.28 |
| Milk protein, % | 3.22 | 3.41 | 3.17 | 0.118 | 0.37 |
| Milk lactose, % | 4.94 | 4.89 | 4.72 | 0.059 | 0.06 |
| Milk total solid, % | 12.7 | 13.3 | 13.0 | 0.331 | 0.53 |
| Milk urea nitrogen, mg/dl | 14.3^b^ | 15.8^b^ | 17.6^a^ | 0.42 | <0.01 |
| Mammary plasma flow, L/d | 18065 | 12507 | 10191 | 1273.4 | <0.01 |

^a-c^ Means within a row that have different superscripts differ significantly (*P*<0.05).

**Table S3.** Summary of the model fitness of GC–TOF/MS analysis about arterial (W) and venous (R) plasma group

| Model | R^2^X^1^ | R^2^Y^2^ | Q^2^ |
| --- | --- | --- | --- |
| W-AH vs. R-AH |  |  |  |
| PCA | 0.54 |  | 0.15 |
| PLS-DA | 0.37 | 0.88 | 0.53 |
| OPLS-DA | 0.37 | 0.88 | 0.56 |
| W-CS vs. R-CS |  |  |  |
| PCA | 0.45 |  | 0.09 |
| PLS-DA | 0.37 | 0.93 | 0.76 |
| OPLS-DA | 0.48 | 0.96 | 0.86 |
| W-RS vs. R-RS |  |  |  |
| PCA | 0.57 |  | 0.11 |
| PLS-DA | 0.45 | 0.95 | 0.61 |
| OPLS-DA | 0.57 | 0.96 | 0.85 |

^*^R^2^X and R^2^Y indicate the cumulative sum of squares of all the X variations and Y variations explained by all extracted components.

^**^Q^2^values display the cumulative percent of the variation in Y and can be used to estimate how well the model predicts the Y.

**Table S4.** The linear correlation (R) and its P-value between the animal performance and mammary uptake of metabolites

|  | 4-Hydroxybutyrate | α-Ketoisocaproic acid | Azelaic acid | β-Mannosylglycerate | Citric acid | D-Galacturonic acid | Diglycerol | D-Talose | Fructose | Glycolic acid | Mannitol | Methylmalonic acid | Salicylic acid | Sorbose | Stearic acid | Tagatose | Threitol | Threonine |
| --- | --- | --- | --- | --- | --- | --- | --- | --- | --- | --- | --- | --- | --- | --- | --- | --- | --- | --- |
| **R-value** |  |  |  |  |  |  |  |  |  |  |  |  |  |  |  |  |  |  |
| Milk yield | 0.54 | 0.63 | -0.60 | -0.45 | 0.57 | 0.52 | 0.69 | 0.70 | 0.72 | 0.42 | -0.56 | -0.47 | -0.54 | 0.60 | 0.62 | 0.45 | -0.52 | 0.52 |
| Feed intake | 0.61 | 0.62 | -0.39 | -0.60 | 0.75 | 0.64 | 0.69 | 0.69 | 0.71 | 0.57 | -0.28 | 0.05 | -0.55 | 0.59 | 0.57 | 0.55 | -0.35 | 0.10 |
| Feed efficiency | 0.26 | 0.34 | -0.48 | -0.28 | 0.18 | 0.24 | 0.46 | 0.50 | 0.45 | 0.11 | -0.49 | -0.52 | -0.32 | 0.34 | 0.46 | 0.19 | -0.40 | 0.52 |
| Protein yield | 0.56 | 0.51 | -0.54 | -0.41 | 0.51 | 0.54 | 0.62 | 0.64 | 0.66 | 0.37 | -0.44 | -0.51 | -0.46 | 0.57 | 0.53 | 0.30 | -0.43 | 0.49 |
| Fat yield | 0.34 | 0.40 | -0.24 | -0.11 | 0.20 | 0.29 | 0.41 | 0.37 | 0.31 | 0.03 | -0.45 | -0.26 | -0.20 | 0.24 | 0.35 | 0.13 | -0.43 | 0.15 |
| Lactose yield | 0.54 | 0.65 | -0.57 | -0.42 | 0.54 | 0.50 | 0.68 | 0.68 | 0.71 | 0.41 | -0.56 | -0.48 | -0.50 | 0.60 | 0.60 | 0.45 | -0.52 | 0.55 |
| Total solid yield | 0.53 | 0.58 | -0.51 | -0.35 | 0.47 | 0.49 | 0.64 | 0.63 | 0.62 | 0.31 | -0.54 | -0.46 | -0.44 | 0.52 | 0.55 | 0.34 | -0.51 | 0.44 |
| Protein | -0.04 | -0.38 | 0.22 | 0.26 | -0.26 | -0.10 | -0.32 | -0.31 | -0.28 | -0.20 | 0.35 | -0.13 | 0.30 | -0.18 | -0.35 | -0.38 | 0.26 | -0.05 |
| Fat | -0.40 | -0.46 | 0.50 | 0.72 | -0.58 | -0.51 | -0.66 | -0.72 | -0.71 | -0.53 | 0.26 | 0.09 | 0.55 | -0.56 | -0.60 | -0.50 | 0.23 | -0.33 |
| Lactose | 0.44 | 0.54 | -0.22 | -0.30 | 0.27 | 0.32 | 0.50 | 0.48 | 0.48 | 0.27 | -0.35 | -0.24 | -0.19 | 0.41 | 0.40 | 0.35 | -0.27 | 0.38 |
| Total solid | -0.27 | -0.40 | 0.43 | 0.62 | -0.51 | -0.39 | -0.55 | -0.60 | -0.59 | -0.45 | 0.26 | 0.00 | 0.50 | -0.45 | -0.52 | -0.46 | 0.21 | -0.23 |
| **P-value** |  |  |  |  |  |  |  |  |  |  |  |  |  |  |  |  |  |  |
| Milk yield | <0.01 | <0.01 | <0.01 | 0.02 | <0.01 | <0.01 | <0.01 | <0.01 | <0.01 | 0.03 | <0.01 | 0.01 | <0.01 | <0.01 | <0.01 | 0.02 | <0.01 | <0.01 |
| Feed intake | <0.01 | <0.01 | 0.04 | <0.01 | <0.01 | <0.01 | <0.01 | <0.01 | <0.01 | <0.01 | 0.15 | 0.80 | <0.01 | <0.01 | <0.01 | <0.01 | 0.08 | 0.63 |
| Feed efficiency | 0.20 | 0.08 | 0.01 | 0.15 | 0.36 | 0.24 | 0.02 | <0.01 | 0.02 | 0.57 | <0.01 | <0.01 | 0.10 | 0.08 | 0.01 | 0.33 | 0.04 | <0.01 |
| Protein yield | <0.01 | <0.01 | <0.01 | 0.03 | <0.01 | <0.01 | <0.01 | <0.01 | <0.01 | 0.06 | 0.02 | <0.01 | 0.02 | <0.01 | <0.01 | 0.12 | 0.02 | <0.01 |
| Fat yield | 0.08 | 0.04 | 0.24 | 0.60 | 0.31 | 0.14 | 0.03 | 0.06 | 0.12 | 0.87 | 0.02 | 0.18 | 0.31 | 0.24 | 0.08 | 0.51 | 0.02 | 0.47 |
| Lactose yield | <0.01 | <0.01 | <0.01 | 0.03 | <0.01 | <0.01 | <0.01 | <0.01 | <0.01 | 0.03 | <0.01 | 0.01 | <0.01 | <0.01 | <0.01 | 0.02 | <0.01 | <0.01 |
| Total solid yield | <0.01 | <0.01 | <0.01 | 0.07 | 0.01 | 0.01 | <0.01 | <0.01 | <0.01 | 0.12 | <0.01 | 0.02 | 0.02 | <0.01 | <0.01 | 0.08 | <0.01 | 0.02 |
| Protein | 0.86 | 0.05 | 0.27 | 0.18 | 0.18 | 0.61 | 0.10 | 0.12 | 0.15 | 0.32 | 0.02 | 0.18 | 0.31 | 0.37 | 0.07 | 0.05 | 0.19 | 0.79 |
| Fat | 0.04 | 0.05 | <0.01 | <0.01 | <0.01 | <0.01 | <0.01 | <0.01 | <0.01 | <0.01 | 0.19 | 0.64 | <0.01 | <0.01 | <0.01 | <0.01 | 0.25 | 0.09 |
| Lactose | 0.02 | <0.01 | 0.27 | 0.13 | 0.17 | 0.10 | <0.01 | 0.01 | 0.01 | 0.17 | 0.08 | 0.23 | 0.33 | 0.03 | 0.04 | 0.08 | 0.17 | 0.05 |
| Total solid | 0.17 | 0.04 | 0.02 | <0.01 | <0.01 | 0.04 | <0.01 | <0.01 | <0.01 | 0.02 | 0.20 | 1.00 | <0.01 | 0.02 | <0.01 | 0.02 | 0.28 | 0.25 |

**Table S5.** The linear correlation (R-value) and its P-value between the animal performance and mammary clearance of metabolites

|  | 3-Aminoisobutyric acid | 3-Hydroxynorvaline | 4-Hydroxybutyrate | α-Ketoisocaproic acid | Azelaic acid | β-Mannosylglycerate | Citric acid | D-Galacturonic acid | Diglycerol | D-Talose | Fructose | Glycolic acid | Maleamate | Myo-inositol | Oleic acid | Ornithine | Phenylacetic acid | Phenylalanine | Salicylic acid | Sorbose | Stearic acid | Tyrosine |
| --- | --- | --- | --- | --- | --- | --- | --- | --- | --- | --- | --- | --- | --- | --- | --- | --- | --- | --- | --- | --- | --- | --- |
| **R-value** |  |  |  |  |  |  |  |  |  |  |  |  |  |  |  |  |  |  |  |  |  |  |
| Milk yield | -0.37 | 0.61 | 0.66 | 0.54 | -0.60 | -0.65 | 0.63 | 0.54 | 0.68 | 0.69 | 0.67 | 0.54 | -0.41 | -0.65 | -0.67 | -0.33 | -0.39 | -0.24 | -0.43 | 0.51 | 0.54 | -0.64 |
| Feed intake | -0.42 | 0.65 | 0.75 | 0.53 | -0.44 | -0.74 | 0.65 | 0.63 | 0.68 | 0.67 | 0.57 | 0.53 | -0.55 | -0.74 | -0.63 | -0.36 | -0.47 | -0.35 | -0.54 | 0.40 | 0.47 | -0.65 |
| Feed efficiency | -0.28 | 0.33 | 0.34 | 0.30 | -0.48 | -0.41 | 0.33 | 0.28 | 0.45 | 0.50 | 0.44 | 0.30 | -0.24 | -0.41 | -0.47 | -0.24 | -0.28 | -0.19 | -0.28 | 0.33 | 0.39 | -0.44 |
| Protein yield | -0.18 | 0.60 | 0.63 | 0.40 | -0.48 | -0.55 | 0.58 | 0.56 | 0.61 | 0.64 | 0.63 | 0.49 | -0.30 | -0.55 | -0.51 | -0.31 | -0.27 | -0.15 | -0.34 | 0.52 | 0.46 | -0.57 |
| Fat yield | -0.05 | 0.31 | 0.33 | 0.36 | -0.15 | -0.30 | 0.18 | 0.31 | 0.42 | 0.37 | 0.25 | 0.12 | -0.09 | -0.30 | -0.35 | 0.05 | -0.09 | 0.00 | -0.27 | 0.16 | 0.34 | -0.23 |
| Lactose yield | -0.35 | 0.58 | 0.64 | 0.56 | -0.55 | -0.61 | 0.62 | 0.53 | 0.67 | 0.68 | 0.68 | 0.55 | -0.38 | -0.61 | -0.63 | -0.28 | -0.34 | -0.18 | -0.42 | 0.53 | 0.53 | -0.61 |
| Total solid yield | -0.23 | 0.55 | 0.59 | 0.50 | -0.46 | -0.55 | 0.51 | 0.51 | 0.63 | 0.62 | 0.58 | 0.43 | -0.30 | -0.55 | -0.56 | -0.21 | -0.28 | -0.14 | -0.39 | 0.45 | 0.49 | -0.53 |
| Protein | 0.48 | -0.19 | -0.21 | -0.40 | 0.35 | 0.40 | -0.22 | -0.13 | -0.31 | -0.31 | -0.19 | -0.21 | 0.33 | 0.40 | 0.52 | 0.19 | 0.41 | 0.32 | 0.34 | -0.06 | -0.28 | 0.31 |
| Fat | 0.57 | -0.58 | -0.63 | -0.40 | 0.61 | 0.76 | -0.64 | -0.53 | -0.62 | -0.70 | -0.61 | -0.57 | 0.62 | 0.76 | 0.63 | 0.59 | 0.62 | 0.52 | 0.51 | -0.43 | -0.44 | 0.74 |
| Lactose | -0.25 | 0.31 | 0.44 | 0.52 | -0.20 | -0.38 | 0.38 | 0.36 | 0.49 | 0.48 | 0.49 | 0.37 | -0.24 | -0.38 | -0.36 | -0.05 | -0.16 | -0.03 | -0.32 | 0.41 | 0.36 | -0.39 |
| Total solid | 0.56 | -0.48 | -0.50 | -0.36 | 0.56 | 0.67 | -0.53 | -0.41 | -0.52 | -0.59 | -0.48 | -0.47 | 0.56 | 0.67 | 0.60 | 0.53 | 0.60 | 0.51 | 0.46 | -0.31 | -0.38 | 0.63 |
| **P-value** |  |  |  |  |  |  |  |  |  |  |  |  |  |  |  |  |  |  |  |  |  |  |
| Milk yield | 0.06 | <0.01 | <0.01 | <0.01 | <0.01 | <0.01 | <0.01 | <0.01 | <0.01 | <0.01 | <0.01 | <0.01 | 0.03 | <0.01 | <0.01 | 0.09 | 0.04 | 0.23 | 0.02 | <0.01 | <0.01 | <0.01 |
| Feed intake | 0.03 | <0.01 | <0.01 | <0.01 | 0.02 | <0.01 | <0.01 | <0.01 | <0.01 | <0.01 | <0.01 | <0.01 | <0.01 | <0.01 | <0.01 | 0.06 | 0.01 | 0.07 | <0.01 | 0.04 | 0.01 | <0.01 |
| Feed efficiency | 0.16 | 0.09 | 0.08 | 0.13 | 0.01 | 0.03 | 0.09 | 0.15 | 0.02 | <0.01 | 0.02 | 0.13 | 0.24 | 0.03 | 0.01 | 0.22 | 0.15 | 0.35 | 0.17 | 0.10 | 0.04 | 0.02 |
| Protein yield | 0.36 | <0.01 | <0.01 | 0.04 | 0.01 | <0.01 | <0.01 | <0.01 | <0.01 | <0.01 | <0.01 | <0.01 | 0.12 | <0.01 | <0.01 | 0.12 | 0.17 | 0.46 | 0.08 | <0.01 | 0.02 | <0.01 |
| Fat yield | 0.82 | 0.12 | 0.09 | 0.07 | 0.45 | 0.13 | 0.38 | 0.11 | 0.03 | 0.06 | 0.21 | 0.55 | 0.67 | 0.13 | 0.07 | 0.81 | 0.67 | 0.99 | 0.17 | 0.42 | 0.08 | 0.25 |
| Lactose yield | 0.08 | <0.01 | <0.01 | <0.01 | <0.01 | <0.01 | <0.01 | <0.01 | <0.01 | <0.01 | <0.01 | <0.01 | 0.05 | <0.01 | <0.01 | 0.16 | 0.08 | 0.37 | 0.03 | <0.01 | <0.01 | <0.01 |
| Total solid yield | 0.24 | <0.01 | <0.01 | <0.01 | 0.02 | <0.01 | <0.01 | <0.01 | <0.01 | <0.01 | <0.01 | 0.02 | 0.13 | <0.01 | <0.01 | 0.30 | 0.17 | 0.50 | 0.05 | 0.02 | <0.01 | <0.01 |
| Protein | 0.01 | 0.34 | 0.29 | 0.04 | 0.07 | 0.04 | 0.26 | 0.52 | 0.11 | 0.12 | 0.33 | 0.30 | 0.10 | 0.04 | <0.01 | 0.34 | 0.04 | 0.11 | 0.08 | 0.77 | 0.16 | 0.12 |
| Fat | <0.01 | <0.01 | <0.01 | 0.04 | <0.01 | <0.01 | <0.01 | <0.01 | <0.01 | <0.01 | <0.01 | <0.01 | <0.01 | <0.01 | <0.01 | <0.01 | <0.01 | <0.01 | <0.01 | 0.02 | 0.02 | <0.01 |
| Lactose | 0.21 | 0.11 | 0.02 | <0.01 | 0.32 | 0.05 | 0.05 | 0.06 | <0.01 | 0.01 | <0.01 | 0.05 | 0.22 | 0.05 | 0.06 | 0.79 | 0.44 | 0.89 | 0.10 | 0.03 | 0.06 | 0.04 |
| Total solid | <0.01 | 0.01 | <0.01 | 0.07 | <0.01 | <0.01 | <0.01 | 0.03 | <0.01 | <0.01 | 0.01 | 0.01 | <0.01 | <0.01 | <0.01 | <0.01 | <0.01 | <0.01 | 0.02 | 0.12 | 0.05 | <0.01 |


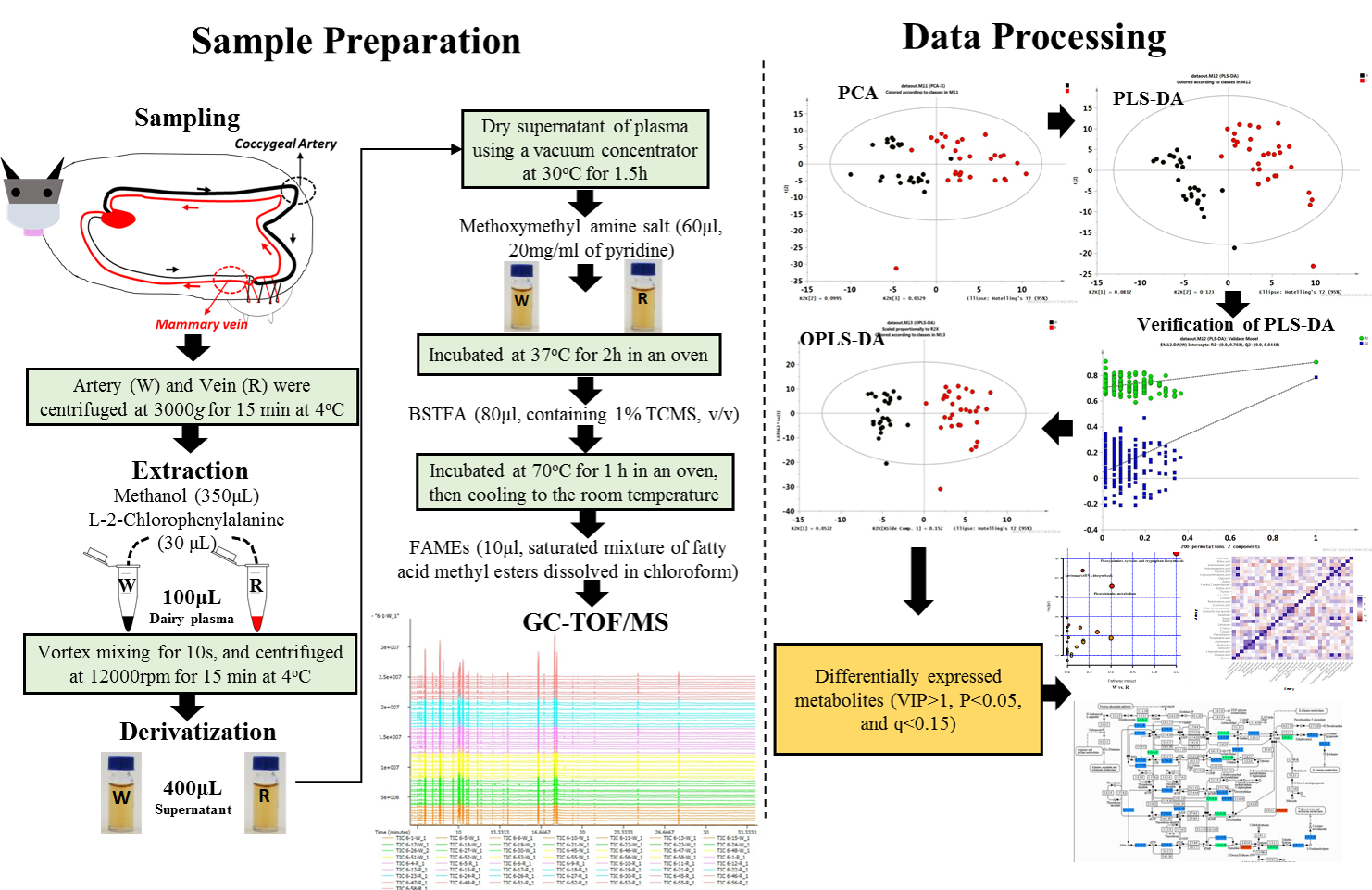


**Figure S1.** The first step in metabolic workflow (left): sample preparation in the experimental design according to the protocol for GC-TOF/MS analysis. The second step in metabolic workflow (right): GC/MS data acquisition and processing using PCA, PLS-DA, verification of PLS-DA, and OPLS-DA. The significantly different changed metabolites (VIP > 1 and p < 0.05) were filtered out between arterial (W) and venous (R) plasma.

**
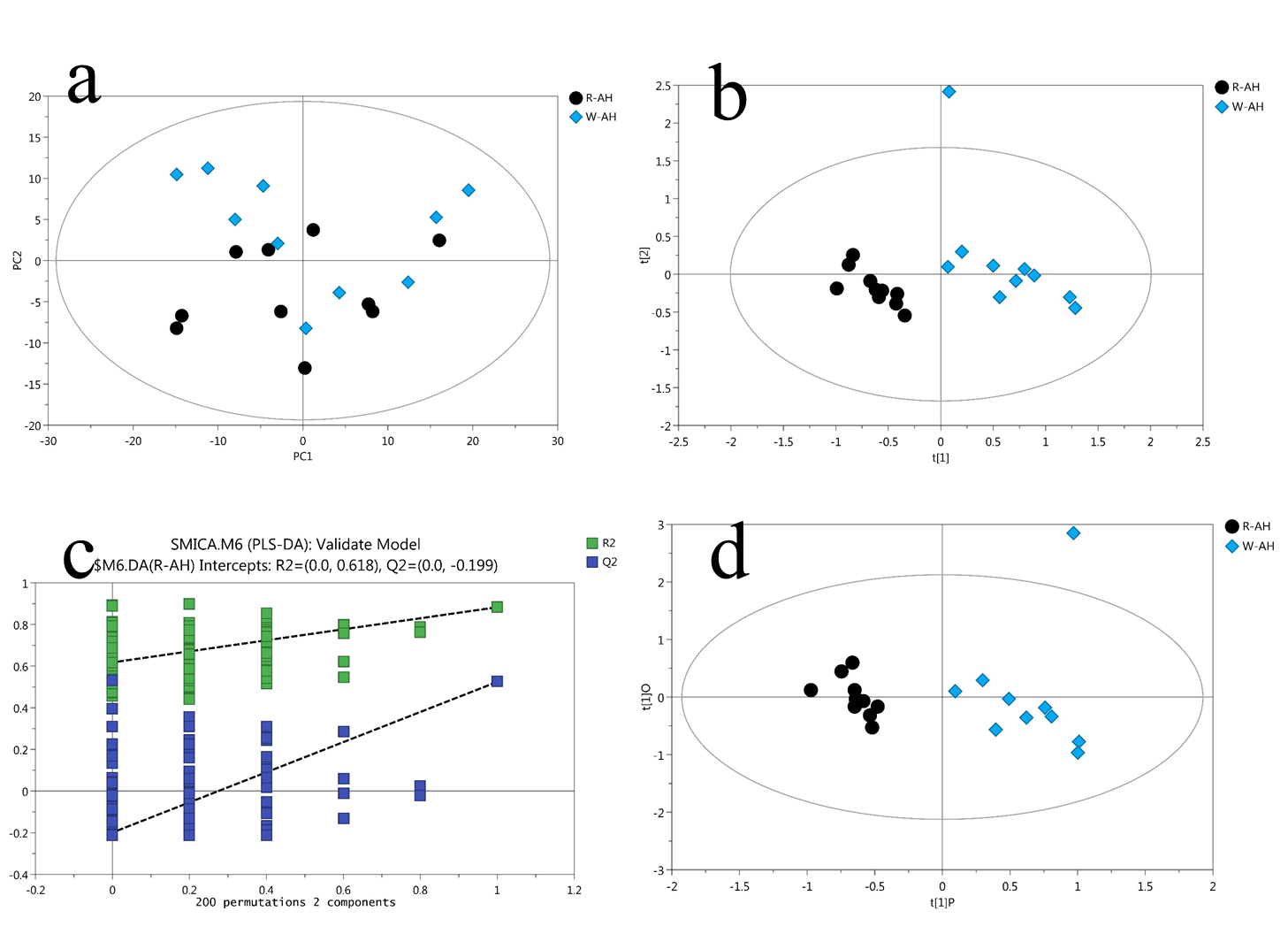
**

**Figure S2.** Score plot of PCA (a), PLS-DA (b), verification of PLS-DA (c), and OPLS-DA (d) model obtained between W-AH and R-AH.


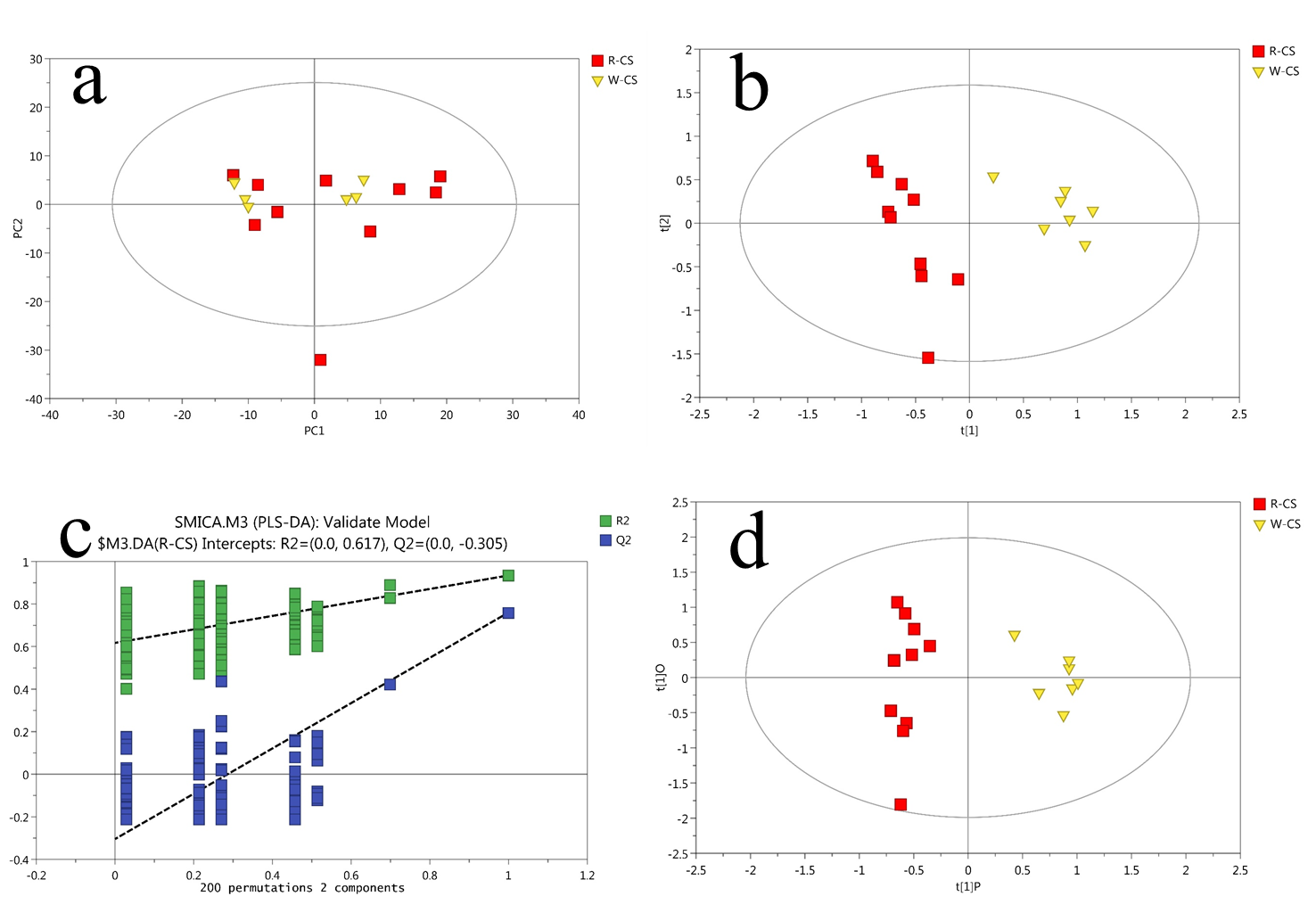


**Figure S3.** Score plot of PCA (a), PLS-DA (b), verification of PLS-DA (c), and OPLS-DA (d) model obtained between W-CS and R-CS.


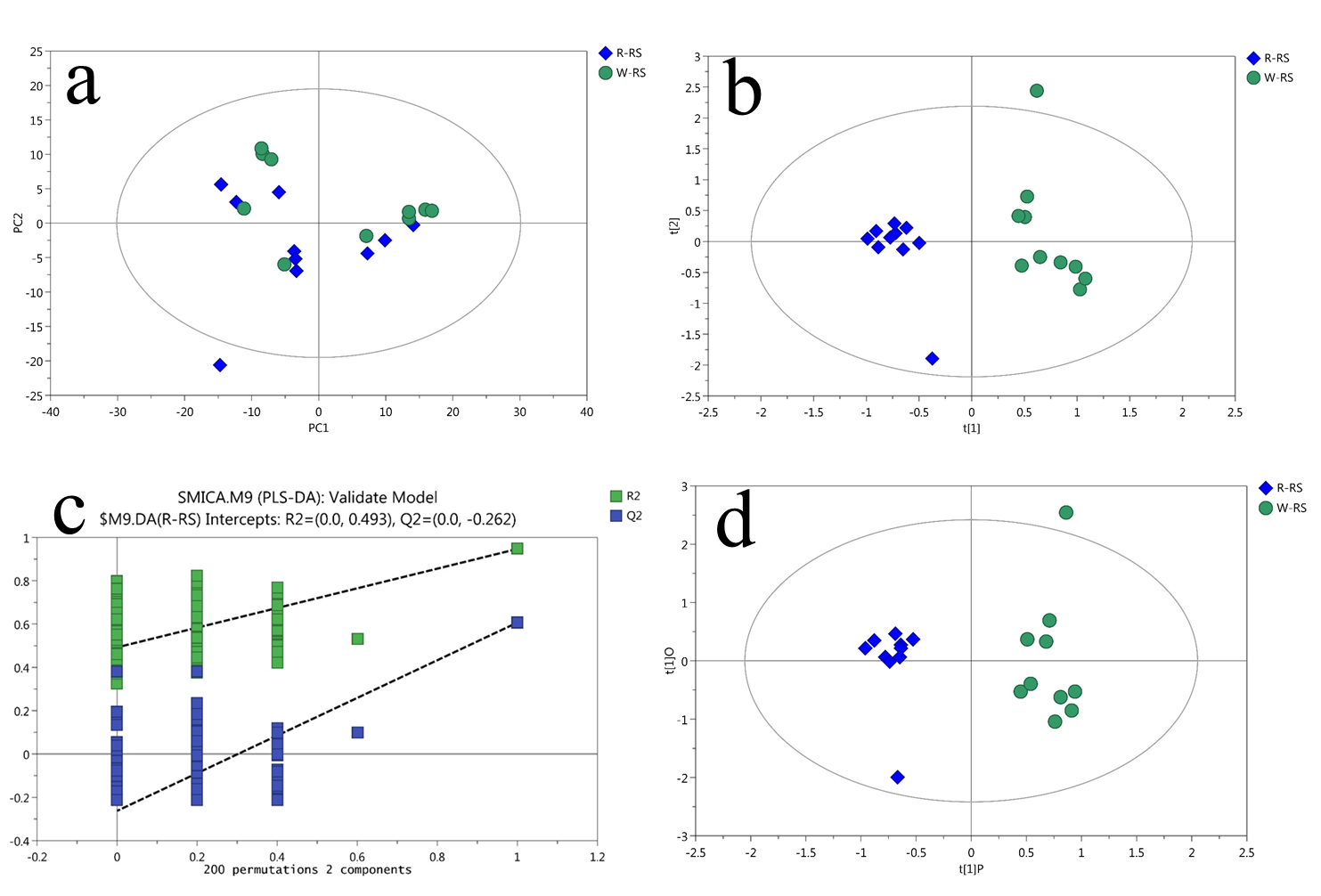


**Figure S4.** Score plot of PCA (a), PLS-DA (b), verification of PLS-DA (c), and OPLS-DA (d) model obtained between W-RS and R-RS.
